# Colour rearrangement for dipole showers

**DOI:** 10.1140/epjc/s10052-018-6070-z

**Published:** 2018-07-26

**Authors:** Johannes Bellm

**Affiliations:** Theoretical Particle Physics, Department of Astronomy and Theoretical Physics, Lund University, Lund, Sweden

## Abstract

We present an algorithm to rearrange the colour chains of dipole showers in the shower process according to the colour amplitudes of a simple matrix element. We implement the procedure in the dipole shower of Herwig and show comparisons to data.

## Introduction

One of the main ingredients to describe data at collider energies in event generators like [1–5] are parton showers. In a probabilistic picture emissions off partons are produced according to probability distributions derived from the collinear and/or soft limit of matrix elements.

Aside from angular ordered shower approximations [6] so-called dipole showers [7] are implemented, as the divergent structure of QCD amplitude can be reproduced in dipole-like emissions without double counting the soft wide-angle emissions. Prominent examples are e.g. the Ariadne shower [5] or parton showers based on Catani Seymour (CS) dipole subtraction [8] earlier introduced for numerical calculation of next-to-leading order (NLO) corrections. CS showers are implemented in Dinsdale et al. [9], Schumann and Krauss [10] and Plätzer and Gieseke [11]. Further developments have been introduced in Höche and Prestel [12] with the recent inclusion of collinear parts of the trilinear NLO splitting in Höche et al. [13] and Höche and Prestel [14] and for antenna showers in Li and Skands [15]. Various methods have been introduced to correct the showering process with matrix element corrections up to a finite number of legs at LO and NLO. These will not be discussed here. In this paper, we concentrate on the colour assignment of this kind of parton shower algorithms and implement an algorithm to rearrange the colour structure in the showering process. Methods to correct the shower emissions with subleading $N_C$ effects have been discussed in Plätzer and Sjödahl [16] and Nagy and Soper [17]. In this article, we discuss the possibility of shower emissions resulting in unfavoured colour configurations and introduce an algorithm to correct with simple LO matrix elements.

It is well known that QCD amplitudes can be decomposed into colour structures [18, 19]. Further colour bases and simplifications have been developed to calculate multileg amplitudes efficiently [20, 21]. For event generation bases like Colourflow [20] or Trace [18, 19] bases have the nice feature, that in the large $N_C$ limit ($N_C$ being the number of colours) the flows or traces can be interpreted as dipole chains/colour lines/strings. The starting conditions of a parton shower process and later the final conditions of the hadronisation process depend on the assignment of colour structures [20, 22, 23]. Event generators keep record of these colour lines and form either clusters of colour connected partons [1, 2, 4] or the lines themselves are interpreted as strings as in Sjöstrand et al. [3].

The maximally helicity violating amplitude with one quark anti-quark pair ($q{\bar{q}}$) and *n*-gluons can be written as (e.g. [24]),

1 $$\begin{aligned} \sum _{col.} \Vert A_{1 \ldots n}\Vert ^2 = \sum _{\{1,2 \ldots n\}} \frac{ {\mathcal {F}}}{(p_q1)(12) \ldots (np_{{\bar{q}}})} + \frac{1}{N_C^2} (interf.) \nonumber \\ \end{aligned}$$

where ${\mathcal {F}}$ is a kinematic factor and $(i j)=2p_i\cdot p_j$ are products of the momenta of gluons and quarks. More general helicity structures are more complicated. The sum on the right-hand side of Eq. (1) includes all permutations of the *n*-gluons and compared to QED the interference is suppressed by a factor of $N_C^{2}$. With the suppression of interference terms the dominating term in the sum of Eq. (1) is the permutation with minimised numerator. In the string picture, this is the shortest string. This chain of dipoles can then be interpreted to have a stringlike behaviour [24].

## Dipole like showers

In this section we summarize the algorithm implemented in Herwig [11, 25] emphasising the colour chain structure and allowing our language to cover other dipole-like shower and hadronisation procedures.

*Starting the shower* In dipole like showers usually, colour chains are assigned before the shower is allowed to evolve the event. A chain contains either one $q{\bar{q}}$-pair and *n*-gluons or only gluons. A chain is then given by $q - g_1-g_2- \cdots -g_n-{\bar{q}}$, where a gluon contains both colour and anti-colour and is, therefore, a member of two dipoles. In the case with only two gluons – e.g. higgs production – there are two colour dipoles between the gluons. In addition to the assignment of colour another initial condition is the chosen starting scale.

*While showering* These chains can then radiate gluons or break by a gluon splitting into a quark anti-quark state, $g\rightarrow q{\bar{q}}$. This is done in a competing Sudakov veto algorithm. Here all dipoles of the chain produce an evolution scale and the dipole with the largest evolution scale is allowed to emit. The emission creates recoils on the other participants in the chain, either because the spectator used to absorb the recoil is again connected to another dipole or the emitter can have another colour connection to another parton in the chain. If, in our example chain above, *q* radiates with spectator $g_1$ the dipole spanned by $g_1$ and $g_2$ is modified as well. Thus, an emission of a gluon will affect the kinematics of up to four of the resulting dipoles. If a gluon splits into a $q{\bar{q}}$-pair the colour of the dipole chain breaks and the quark carries the colour of the split gluon and the anti-quark gets the anti-colour. Once the emission is performed the chain or two chains is/are evolved further until the shower algorithm is terminated by finding no emission scale above the infrared (IR) cutoff.

*Splitting a dipole* Once a dipole in the chain is identified to be the winning participant, a momentum fraction *z* is chosen according to the functional form of the splitting function. If spin-averaged splitting functions are used, the radiation angle $\phi$ of the emission plane around the dipole axis is chosen randomly on the interval $[0,2\pi )$. For spin-dependent splitting functions, the radiation angle can be biased by the helicity of the emitting parton.

*After the shower* Once the IR cutoff is reached the colour chains are interpreted as colour strings and hardonised in a Lund string model or the remaining gluons are split to break the chains in colour anti-colour $q{\bar{q}}$-pairs which then build the clusters of the cluster models. After forming of clusters/strings the process of colour reconnection can rearrange the constituents of the clusters/strings and in addition cluster fissioning or string breaking happens before cluster masses/string lengths are reached that allows the conversion to hadronic states.

## Colour rearrangement

If we interpret terms as in Eq. (1) as probabilities to choose colour lines for the starting conditions of the showering process, we now want to know what happens to the colour structure after emitting off a dipole in a given dipole chain The goal is to construct an algorithm to correct of possible miss-ordered colour structures with simple factories MEs as it shall become clear in the following.

Emitting a gluon $g_a$ from dipole $g_1 - g_2$^1^ leads us to:

2 $$\begin{aligned}&q -g_1 = g_2 - g_3 - g_4 - \cdots - g_n - {\bar{q}} \\&(a)\rightarrow q - g_1' - g_a - g_2' - g_3 - g_4 - \cdots - g_n - {\bar{q}} \nonumber \\&(b)\rightarrow q - g_a - g_1' - g_2' - g_3 - g_4 - \cdots - g_n - {\bar{q}} \nonumber \\&(c)\rightarrow q - g_1' - g_2' - g_a - g_3 - g_4 - \cdots - g_n - {\bar{q}} \nonumber \end{aligned}$$

Here gluon $g_a$ should be identified as the softer gluon in the splitting of $g_1$ with a spectator $g_2$. Configuration (a) is obtained if the emission angle is such that the softer gluon is ‘in between’ the new $g_1'$ and $g_2'$. (b) is obtained when $g_a$ is closer to the quark $q_p$ than $g_1$. (c) is a configuration that is not obtained by CS showers but can happen if the emitter-spectator relation is not clear as in Ariadne. In the colourflow picture (a), (b) and (c) in Eq. (2) correspond to permutations of inner gluons and the weights of assigning the colour lines depends on the full chain. If we would start the shower from the configuration received after emission we would assign the colours according to the weights in the colour representation. Here the emitted gluon ‘feels’ the nearby gluon and the colours are arranged accordingly. The independent dipole in a chain has no possibility to distinguish a preferred direction in terms of colour amplitudes.

**Fig. 1:**
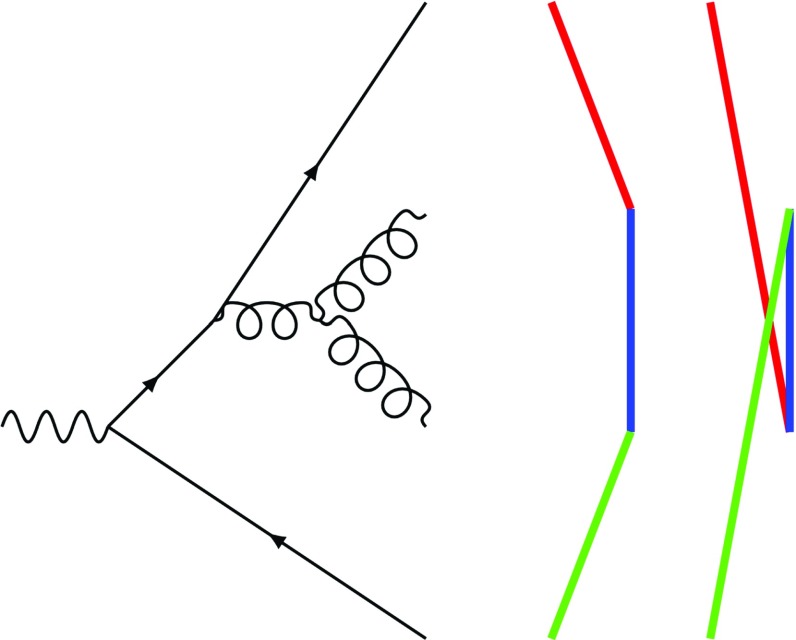
Simple process with three colour dipoles. This process is used to calculate the weight for the colour rearrangement

In the physical picture where most of the emission of $$g_1$$ is in the angle opened by $$g_1-g_2$$ or possibly but suppressed closer (in this example) to the quark we can assume a shielding of colours of dipoles $$g_2' - g_3 - g_4 - \cdots $$. Then these distant dipoles have little effect on the emission off $$g_1$$. The colour connected quark *q*, however, is close to the colour line of gluon $$g_2'$$. In order to construct a weight to distinguish between configurations (a) and (b) we can use the simplest matrix element available that includes three dipoles namely e.g. $$\gamma * \rightarrow u {\bar{u}} g g$$, see Fig. 1. We are only interested in the distinction between colour structure $$q - g_1' - g_a - g_2' - $$ and $$q - g_a - g_1' - g_2' - $$ as the rest of the event remains unchanged. Even the identification of the $${\bar{u}}$$ to represent gluon $$g'_2$$ is a good approximation as its colour charge vanishes in the weight ratio used to decide between the states.

In the actual implementation, we define a phase space point $\Phi$ from three dipoles and incoming beams to deliver the energy needed for the dipole combination. We use MadGraph [26] to generate the process $e^+e^- \rightarrow u {\bar{u}} g g$ and calculate the weights of the squared colour amplitudes *w*(1; 2; 3; 4) = $\mathtt{jamp2[0]}$ and *w*(1; 3; 2; 4) = $\mathtt{jamp2[1]}$ at the given $\Phi$. If a flat random number in [0, 1) is smaller than

$$\begin{aligned} P_{\text {swap}}=\frac{w(1;3;2;4)}{w(1;2;3;4)+w(1;3;2;4)} \end{aligned}$$

we swap the momenta of the gluons $g_2$ and $g_3$ which corresponds to rearranging the colour structure. Note that the weights take into account interferences and parts of off-shell effects but neglect the non-diagonal elements in the colour basis.

As the matrix element is simple and fast to compute we also allow swapping in the chain that was not modified by the last emission, by simply calculating the swapping probability for all neighboring triple dipoles. If the colour chain is already in an order preferred by the matrix element this will not change the probability of having this colour structure, see third comment in Sect. 4, if not the lines will be rearranged to the‘preferred’ order. Preferred is a probabilistic mixture of short or long chains which is now given by $P_{\text {swap}}$ rather than an uncontrolled function of evolution variable and emission angle.

## Comments

We would like to add some comments on the rearrangements:

1. By swapping to the preferred smaller dipole masses we allow fewer emissions in the following, as the dipole phase space is given by the mass of the dipole.
2. Assume a shower produces, for a given phase space point, the preferred colour structure with ME weight $w_1=w(1;2;3;4)$ with probability *a* and the unfavoured colour structure with probability $1-a$. Then a swapping will produce the favoured colour structure as,^2^
  $$\begin{aligned} \left( 1-\frac{w_2}{w_1+w_2}\right) \cdot a + \left( \frac{w_1}{w_1+w_2}\right) \cdot (1-a)=\frac{w_1}{w_1+w_2} \end{aligned}$$
  and similar for the unfavoured colour structure.
3. This method does not need weighted events to correct for the colour assignment.
4. The rearrangement can be performed at any step of the shower and is not restricted to a given multiplicity.
5. A possible failure of the method is the rearrangement to produce dipoles with masses that are too small to create colour singlets that further can decay to mesonic states. We did not yet observe this behaviour.
6. It is anticipated that we can use the same process to rearrange the colours of incoming partons if we do not allow the swapping of final state to initial state momenta. To do so we will in a further publication invert the incoming three-momenta and define all dipole participants as outgoing. As we sum over all helicity combinations this should give the correct weights.
7. Once the method is extended to LHC physics the colour reconnection model needs to be reviewed/retuned as the rearrangement will create another density of cluster masses/strings sizes.
8. Using matrix elements with longer dipole chains e.g. $\gamma * \rightarrow u {\bar{u}} g g g$ to distinguish more permutations of intermediate gluons is part of future work.
9. It is clear that the method can be applied to any kind of dipole like shower e.g. the Sherpa [10], the final state shower of Pythia [3] as well as the Dire shower [12]. In discussions with the authors of these showers it became clear that in the actual implementation the colour assignments in gluons splittings is performed to correct for the symmetric gluon splitting function. The effects of these choices will be subject of future work.

## Results

In order not to bias^3^ the results by tuning we choose to use the tuned values of the improved agular ordered or ${\tilde{Q}}$ shower of Herwig [27]. Further tuning of the shower with the modifications described in this work will improve the description of data but is also able to hide the effects due to rearranging the colours. Namely, parameters controlling the Cluster fission mechanism might allow having similar effects, as the number of particles can be reduced either by splitting clusters less often or, as in this approach, by reducing the average dipole sizes. With the choice to use the value tuned for the ${\tilde{Q}}$ shower two parameters are free. The value of the strong coupling is $\alpha ^{\overline{\mathrm{MS}}}_S(M_Z)=0.118$ and the IR cutoff $\mu$ is varied by 0.6 / 0.8 / 1.0 GeV. We convert the $\overline{\mathrm{MS}}$  value of $\alpha _S$ to the MC scheme (CMW) [28] with the appropriate factor. With these values we show^4^

**Fig. 2:**
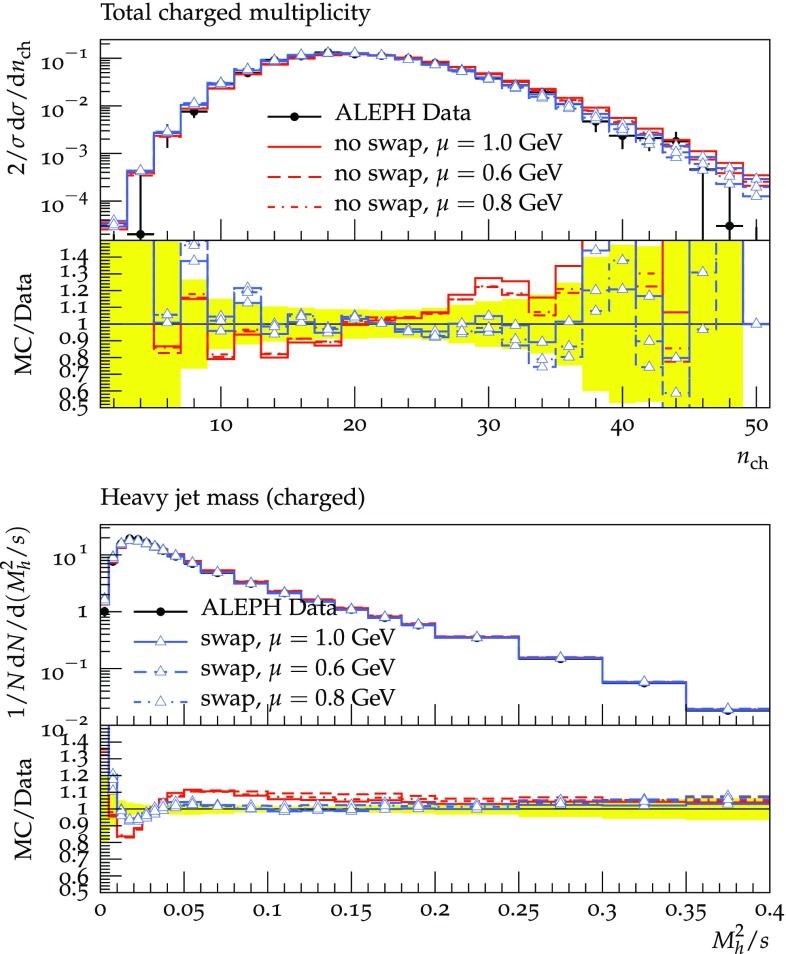
Upper plot: the total charge multiplicity as measured by ALEPH Collaboration [30]. Lower plot: The heavy jet mass measured by ALEPH Collaboration [31]. For simulation setup see Sect. 5

**Fig. 3:**
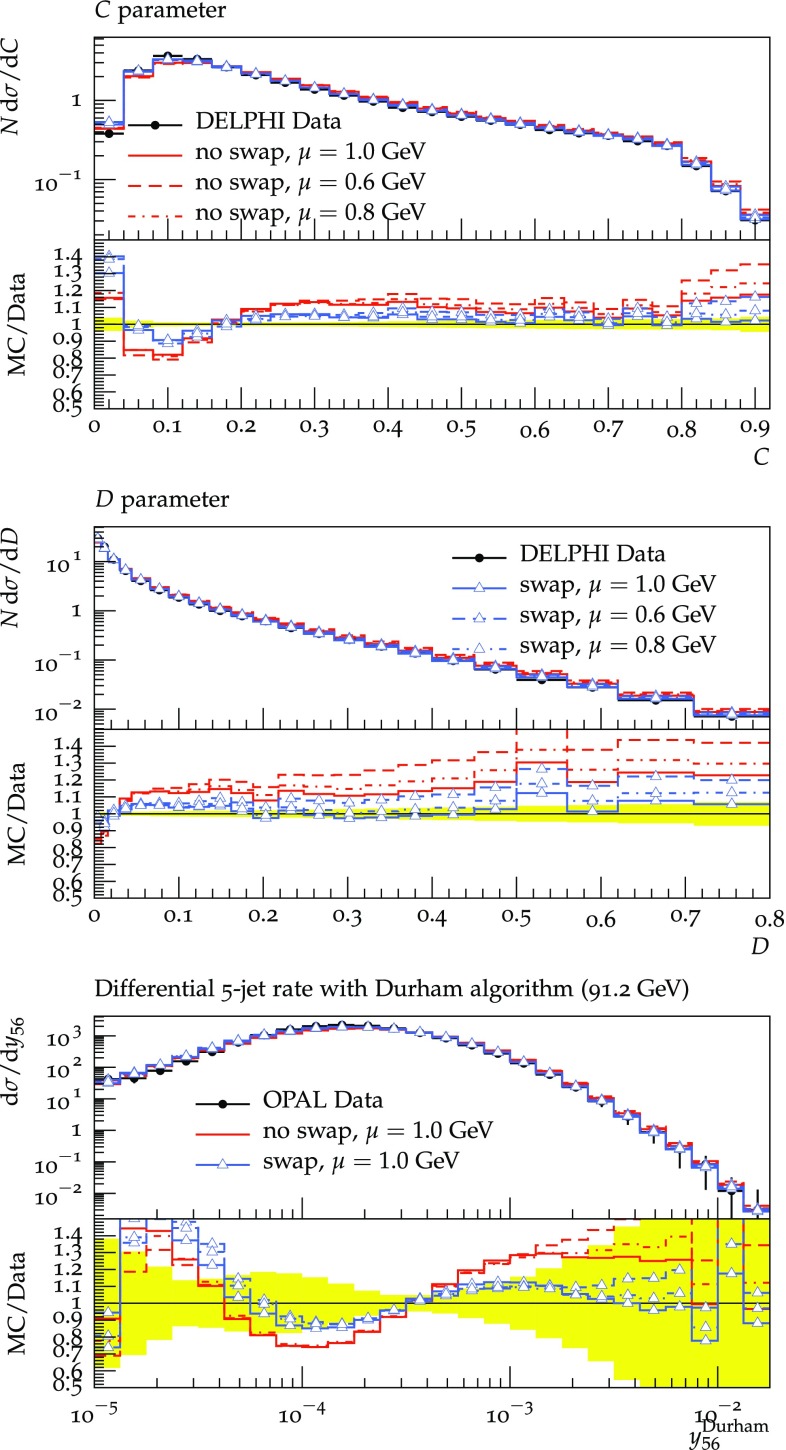
C and D parameter as measured by DELPHI Collaboration [32]. Differential 5-jet rate as measured by JADE, OPAL Collaboration [33]. For details see Sect. 5

- the charge multiplicity as measured in [30], see Fig. 2
- the heavy jet mass from [31], see Fig. 2
- the C- and D-parameter as measured in [32], see Fig. 3
- the five jet rate measured here [33], see Fig. 3

We see large effects (up to $40\%$ for standard LEP observables) and an overall improvement with respect to the standard dipole showering. It is notable that the observables shown here are sensitive to multiple emissions and we have checked that observables sensitive to fewer emissions are in general not described worse. Fully tuned results including $\chi ^2$ comparisons as well as the extension to LHC physics will be discussed in future work.

## Conclusion

In this paper we concentrated on the colour assignment in commonly used dipole-like parton showers. We then developed a method to assign a probability to the rearrangement of colour dipoles. The method allows producing ‘shorter’ dipole chains if the shower falsely produces heavy chains. We finally show example comparison to data and see that not only the rearrangement can have effects of the order of up to $40\%$ in standard observables but also by choosing an independent tune LEP data is better described by the procedure. Various future projects including formal proofs, comparison to resummation and physics application are proposed.

## Footnotes

1 The following argument holds for emissions off the ends of the chains with a less complex structure.

2 Produce favoured and remain and produce unfavoured but change.

3 By assuming an improved coherence picture after rearranging color dipoles this statement might be questionable.

4 For analysing and plotting Rivet [29] was used.
